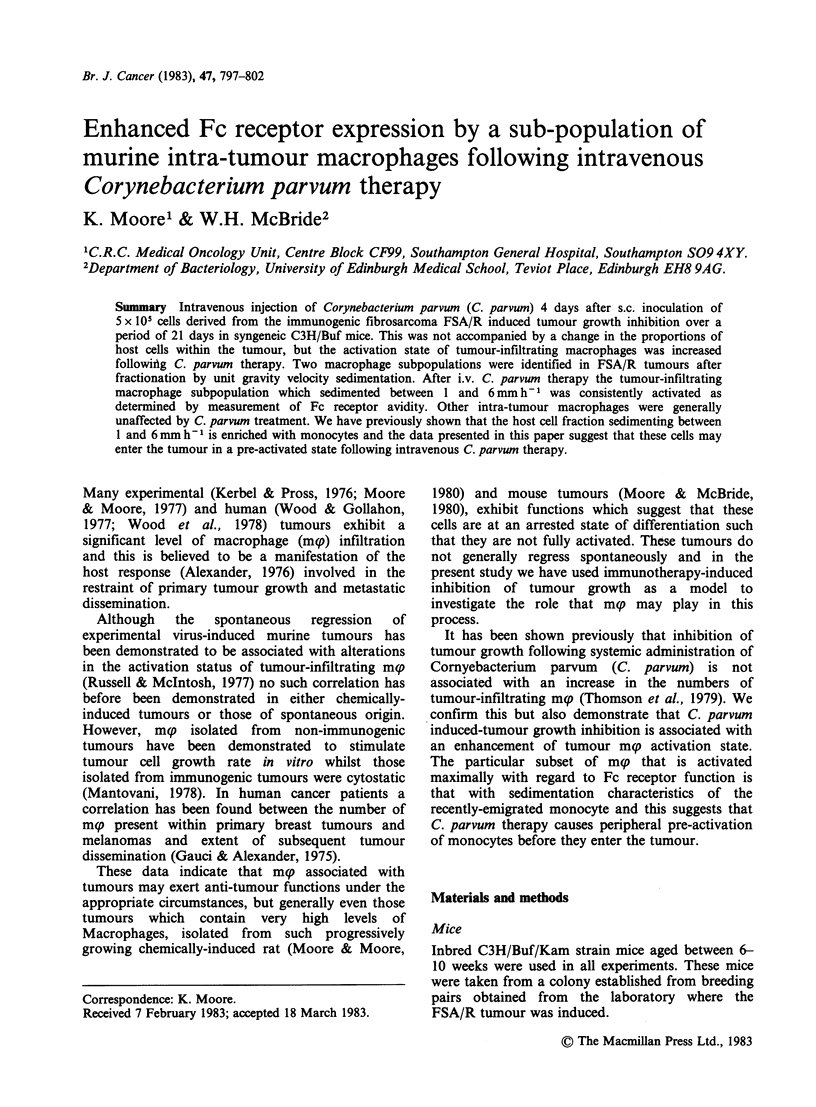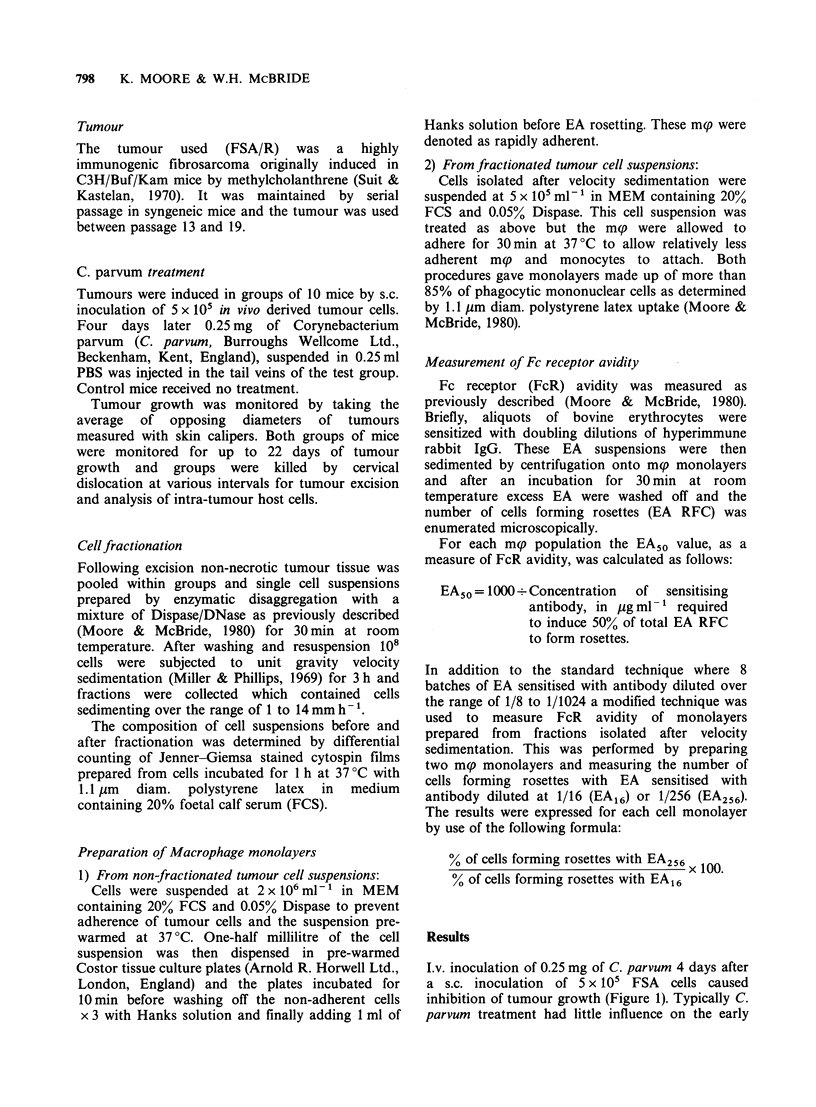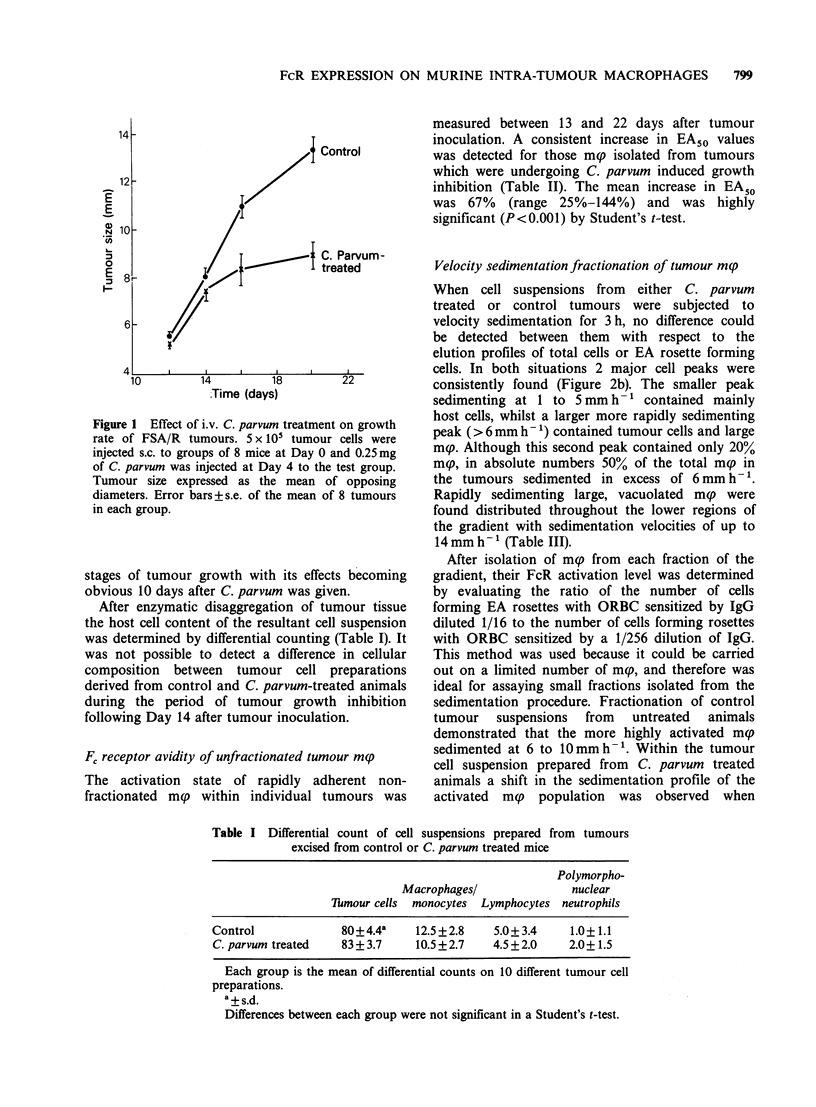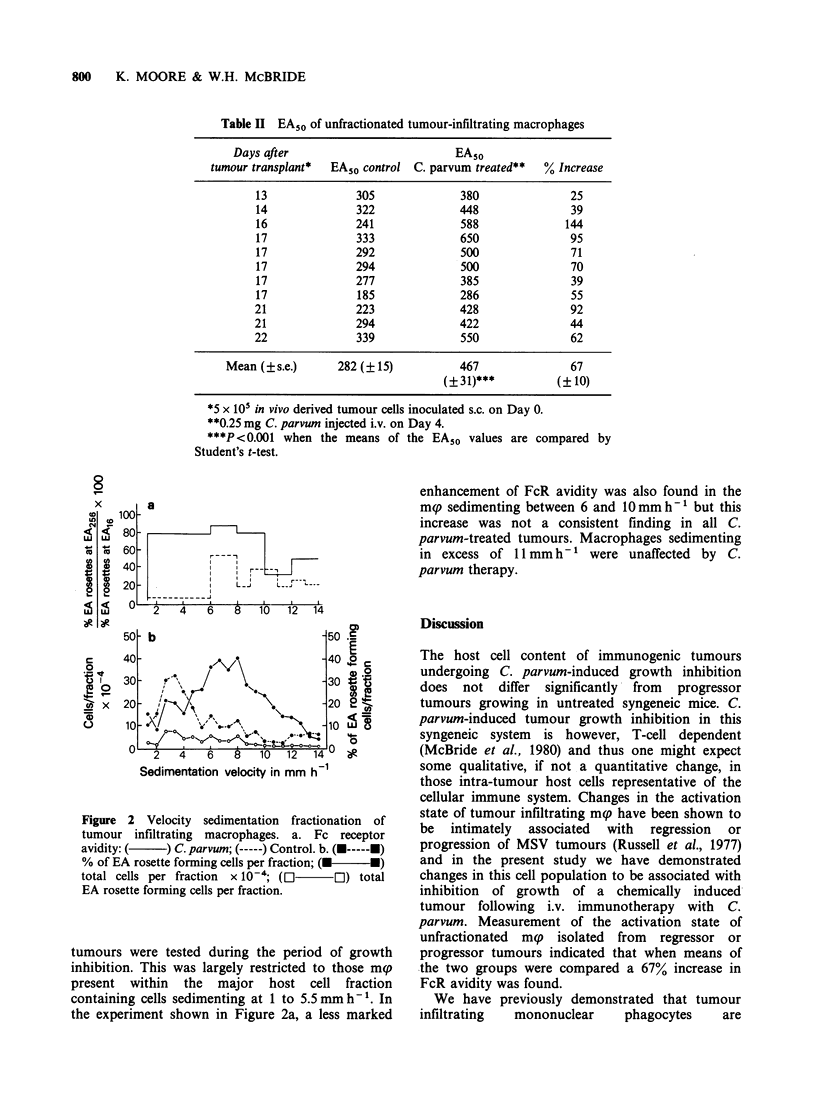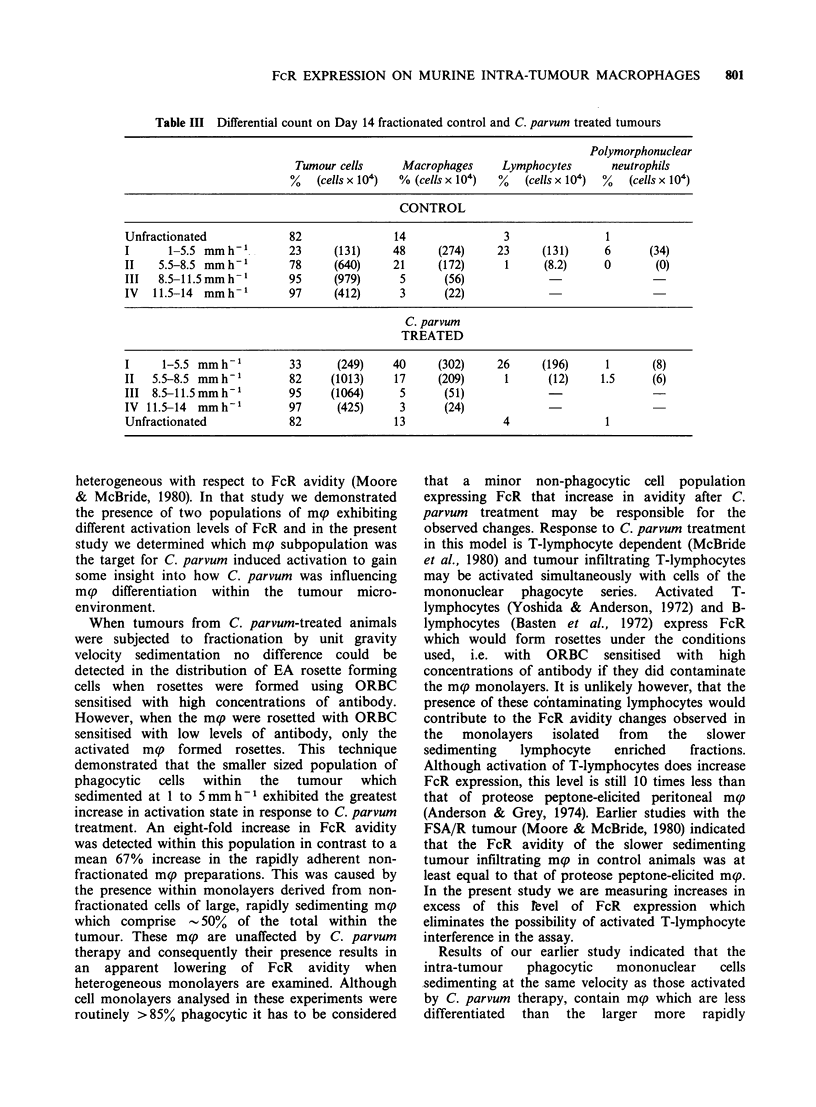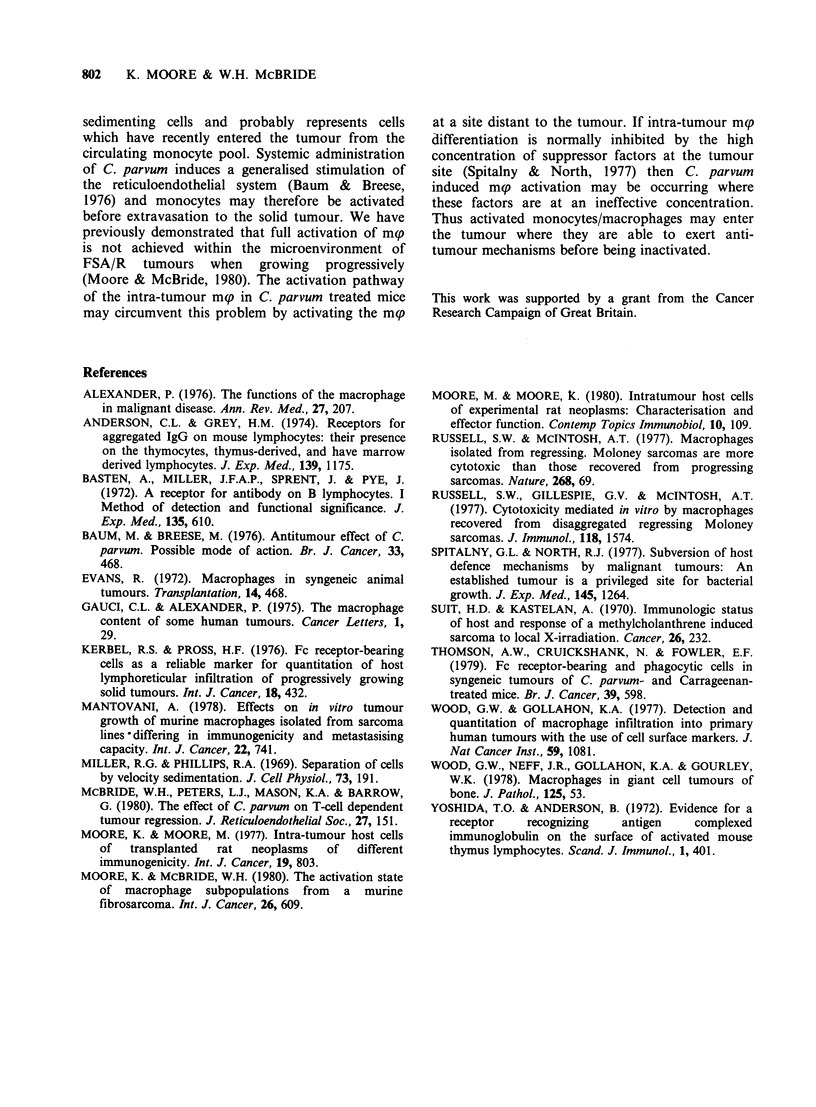# Enhanced Fc receptor expression by a sub-population of murine intra-tumour macrophages following intravenous Corynebacterium parvum therapy.

**DOI:** 10.1038/bjc.1983.133

**Published:** 1983-06

**Authors:** K. Moore, W. H. McBride

## Abstract

Intravenous injection of Corynebacterium parvum (C. parvum) 4 days after s.c. inoculation of 5 X 10(5) cells derived from the immunogenic fibrosarcoma FSA/R induced tumour growth inhibition over a period of 21 days in syngeneic C3H/Buf mice. This was not accompanied by a change in the proportions of host cells within the tumour, but the activation state of tumour-infiltrating macrophages was increased following C. parvum therapy. Two macrophage subpopulations were identified in FSA/R tumours after fractionation by unit gravity velocity sedimentation. After i.v. C. parvum therapy the tumour-infiltrating macrophage subpopulation which sedimented between 1 and 6 mm h-1 was consistently activated as determined by measurement of Fc receptor avidity. Other intra-tumour macrophages were generally unaffected by C. parvum treatment. We have previously shown that the host cell fraction sedimenting between 1 and 6 mm h-1 is enriched with monocytes and the data presented in this paper suggest that these cells may enter the tumour in a pre-activated state following intravenous C. parvum therapy.


*Br. J. Cancer* (1983), **47**, 797–802

### Enhanced Fc receptor expression by a sub-population of murine intra-tumour macrophages following intravenous *Corynebacterium parvum* therapy

K. Moore[1] & W.H. McBride[2]

[1]*C.R.C. Medical Oncology Unit, Centre Block CF99, Southampton General Hospital, Southampton SO9 4XY.*
[2]*Department of Bacteriology, University of Edinburgh Medical School, Teviot Place, Edinburgh EH8 9AG.*

**Summary** Intravenous injection of *Corynebacterium parvum* (*C. parvum*) 4 days after s.c. inoculation of $5\times10^5$ cells derived from the immunogenic fibrosarcoma FSA/R induced tumour growth inhibition over a period of 21 days in syngeneic C3H/Buf mice. This was not accompanied by a change in the proportions of host cells within the tumour, but the activation state of tumour-infiltrating macrophages was increased following *C. parvum* therapy. Two macrophage subpopulations were identified in FSA/R tumours after fractionation by unit gravity velocity sedimentation. After i.v. *C. parvum* therapy the tumour-infiltrating macrophage subpopulation which sedimented between 1 and $6\,mm\,h^{-1}$ was consistently activated as determined by measurement of Fc receptor avidity. Other intra-tumour macrophages were generally unaffected by *C. parvum* treatment. We have previously shown that the host cell fraction sedimenting between 1 and $6\,mm\,h^{-1}$ is enriched with monocytes and the data presented in this paper suggest that these cells may enter the tumour in a pre-activated state following intravenous *C. parvum* therapy.

Many experimental (Kerbel & Pross, 1976; Moore & Moore, 1977) and human (Wood & Gollahon, 1977; Wood *et al.*, 1978) tumours exhibit a significant level of macrophage (m$\varphi$) infiltration and this is believed to be a manifestation of the host response (Alexander, 1976) involved in the restraint of primary tumour growth and metastatic dissemination.

Although the spontaneous regression of experimental virus-induced murine tumours has been demonstrated to be associated with alterations in the activation status of tumour-infiltrating m$\varphi$ (Russell & McIntosh, 1977) no such correlation has before been demonstrated in either chemically-induced tumours or those of spontaneous origin. However, m$\varphi$ isolated from non-immunogenic tumours have been demonstrated to stimulate tumour cell growth rate *in vitro* whilst those isolated from immunogenic tumours were cytostatic (Mantovani, 1978). In human cancer patients a correlation has been found between the number of m$\varphi$ present within primary breast tumours and melanomas and extent of subsequent tumour dissemination (Gauci & Alexander, 1975).

These data indicate that m$\varphi$ associated with tumours may exert anti-tumour functions under the appropriate circumstances, but generally even those tumours which contain very high levels of Macrophages, isolated from such progressively growing chemically-induced rat (Moore & Moore, 1980) and mouse tumours (Moore & McBride, 1980), exhibit functions which suggest that these cells are at an arrested state of differentiation such that they are not fully activated. These tumours do not generally regress spontaneously and in the present study we have used immunotherapy-induced inhibition of tumour growth as a model to investigate the role that m$\varphi$ may play in this process.

It has been shown previously that inhibition of tumour growth following systemic administration of Cornyebacterium parvum (*C. parvum*) is not associated with an increase in the numbers of tumour-infiltrating m$\varphi$ (Thomson *et al.*, 1979). We confirm this but also demonstrate that *C. parvum* induced-tumour growth inhibition is associated with an enhancement of tumour m$\varphi$ activation state. The particular subset of m$\varphi$ that is activated maximally with regard to Fc receptor function is that with sedimentation characteristics of the recently-emigrated monocyte and this suggests that *C. parvum* therapy causes peripheral pre-activation of monocytes before they enter the tumour.

### Materials and methods

### *Mice*

Inbred C3H/Buf/Kam strain mice aged between 6–10 weeks were used in all experiments. These mice were taken from a colony established from breeding pairs obtained from the laboratory where the FSA/R tumour was induced.

Correspondence: K. Moore.
Received 7 February 1983; accepted 18 March 1983.

© The Macmillan Press Ltd., 1983

*Tumour*

The tumour used (FSA/R) was a highly immunogenic fibrosarcoma originally induced in C3H/Buf/Kam mice by methylcholanthrene (Suit & Kastelan, 1970). It was maintained by serial passage in syngeneic mice and the tumour was used between passage 13 and 19.

C. parvum *treatment*

Tumours were induced in groups of 10 mice by s.c. inoculation of $5 \times 10^5$ *in vivo* derived tumour cells. Four days later 0.25 mg of Corynebacterium parvum (*C. parvum,* Burroughs Wellcome Ltd., Beckenham, Kent, England), suspended in 0.25 ml PBS was injected in the tail veins of the test group. Control mice received no treatment.

Tumour growth was monitored by taking the average of opposing diameters of tumours measured with skin calipers. Both groups of mice were monitored for up to 22 days of tumour growth and groups were killed by cervical dislocation at various intervals for tumour excision and analysis of intra-tumour host cells.

*Cell fractionation*

Following excision non-necrotic tumour tissue was pooled within groups and single cell suspensions prepared by enzymatic disaggregation with a mixture of Dispase/DNase as previously described (Moore & McBride, 1980) for 30 min at room temperature. After washing and resuspension $10^8$ cells were subjected to unit gravity velocity sedimentation (Miller & Phillips, 1969) for 3 h and fractions were collected which contained cells sedimenting over the range of 1 to 14 mm $h^{-1}$.

The composition of cell suspensions before and after fractionation was determined by differential counting of Jenner–Giemsa stained cytospin films prepared from cells incubated for 1 h at 37 °C with 1.1 $\mu$m diam. polystyrene latex in medium containing 20% foetal calf serum (FCS).

*Preparation of Macrophage monolayers*

1) *From non-fractionated tumour cell suspensions*:

Cells were suspended at $2 \times 10^6$ ml$^{-1}$ in MEM containing 20% FCS and 0.05% Dispase to prevent adherence of tumour cells and the suspension pre-warmed at 37 °C. One-half millilitre of the cell suspension was then dispensed in pre-warmed Costor tissue culture plates (Arnold R. Horwell Ltd., London, England) and the plates incubated for 10 min before washing off the non-adherent cells ×3 with Hanks solution and finally adding 1 ml of Hanks solution before EA rosetting. These m$\varphi$ were denoted as rapidly adherent.

2) *From fractionated tumour cell suspensions*:

Cells isolated after velocity sedimentation were suspended at $5 \times 10^5$ ml$^{-1}$ in MEM containing 20% FCS and 0.05% Dispase. This cell suspension was treated as above but the m$\varphi$ were allowed to adhere for 30 min at 37 °C to allow relatively less adherent m$\varphi$ and monocytes to attach. Both procedures gave monolayers made up of more than 85% of phagocytic mononuclear cells as determined by 1.1 $\mu$m diam. polystyrene latex uptake (Moore & McBride, 1980).

*Measurement of Fc receptor avidity*

Fc receptor (FcR) avidity was measured as previously described (Moore & McBride, 1980). Briefly, aliquots of bovine erythrocytes were sensitized with doubling dilutions of hyperimmune rabbit IgG. These EA suspensions were then sedimented by centrifugation onto m$\varphi$ monolayers and after an incubation for 30 min at room temperature excess EA were washed off and the number of cells forming rosettes (EA RFC) was enumerated microscopically.

For each m$\varphi$ population the $EA_{50}$ value, as a measure of FcR avidity, was calculated as follows:

$EA_{50} = 1000 \div$ Concentration of sensitising antibody, in $\mu$g ml$^{-1}$ required to induce 50% of total EA RFC to form rosettes.

In addition to the standard technique where 8 batches of EA sensitised with antibody diluted over the range of 1/8 to 1/1024 a modified technique was used to measure FcR avidity of monolayers prepared from fractions isolated after velocity sedimentation. This was performed by preparing two m$\varphi$ monolayers and measuring the number of cells forming rosettes with EA sensitised with antibody diluted at 1/16 ($EA_{16}$) or 1/256 ($EA_{256}$). The results were expressed for each cell monolayer by use of the following formula:

$$\frac{\text{\% of cells forming rosettes with } EA_{256}}{\text{\% of cells forming rosettes with } EA_{16}} \times 100.$$

### Results

I.v. inoculation of 0.25 mg of *C. parvum* 4 days after a s.c. inoculation of $5 \times 10^5$ FSA cells caused inhibition of tumour growth (Figure 1). Typically *C. parvum* treatment had little influence on the early

**Figure 1** Effect of i.v. *C. parvum* treatment on growth rate of FSA/R tumours. $5\times10^5$ tumour cells were injected s.c. to groups of 8 mice at Day 0 and 0.25 mg of *C. parvum* was injected at Day 4 to the test group. Tumour size expressed as the mean of opposing diameters. Error bars ± s.e. of the mean of 8 tumours in each group.

stages of tumour growth with its effects becoming obvious 10 days after *C. parvum* was given.

After enzymatic disaggregation of tumour tissue the host cell content of the resultant cell suspension was determined by differential counting (Table I). It was not possible to detect a difference in cellular composition between tumour cell preparations derived from control and *C. parvum*-treated animals during the period of tumour growth inhibition following Day 14 after tumour inoculation.

### *$F_c$ receptor avidity of unfractionated tumour mφ*

The activation state of rapidly adherent non-fractionated mφ within individual tumours was measured between 13 and 22 days after tumour inoculation. A consistent increase in $EA_{50}$ values was detected for those mφ isolated from tumours which were undergoing *C. parvum* induced growth inhibition (Table II). The mean increase in $EA_{50}$ was 67% (range 25%–144%) and was highly significant ($P<0.001$) by Student's *t*-test.

### *Velocity sedimentation fractionation of tumour mφ*

When cell suspensions from either *C. parvum* treated or control tumours were subjected to velocity sedimentation for 3 h, no difference could be detected between them with respect to the elution profiles of total cells or EA rosette forming cells. In both situations 2 major cell peaks were consistently found (Figure 2b). The smaller peak sedimenting at 1 to 5 mm h$^{-1}$ contained mainly host cells, whilst a larger more rapidly sedimenting peak (>6 mm h$^{-1}$) contained tumour cells and large mφ. Although this second peak contained only 20% mφ, in absolute numbers 50% of the total mφ in the tumours sedimented in excess of 6 mm h$^{-1}$. Rapidly sedimenting large, vacuolated mφ were found distributed throughout the lower regions of the gradient with sedimentation velocities of up to 14 mm h$^{-1}$ (Table III).

After isolation of mφ from each fraction of the gradient, their FcR activation level was determined by evaluating the ratio of the number of cells forming EA rosettes with ORBC sensitized by IgG diluted 1/16 to the number of cells forming rosettes with ORBC sensitized by a 1/256 dilution of IgG. This method was used because it could be carried out on a limited number of mφ, and therefore was ideal for assaying small fractions isolated from the sedimentation procedure. Fractionation of control tumour suspensions from untreated animals demonstrated that the more highly activated mφ sedimented at 6 to 10 mm h$^{-1}$. Within the tumour cell suspension prepared from *C. parvum* treated animals a shift in the sedimentation profile of the activated mφ population was observed when

**Table I** Differential count of cell suspensions prepared from tumours excised from control or *C. parvum* treated mice

| | *Tumour cells* | *Macrophages/ monocytes* | *Lymphocytes* | *Polymorphonuclear neutrophils* |
|---|---|---|---|---|
| Control | 80±4.4[a] | 12.5±2.8 | 5.0±3.4 | 1.0±1.1 |
| *C. parvum* treated | 83±3.7 | 10.5±2.7 | 4.5±2.0 | 2.0±1.5 |

Each group is the mean of differential counts on 10 different tumour cell preparations.

[a] ± s.d.

Differences between each group were not significant in a Student's *t*-test.

**Table II** $EA_{50}$ of unfractionated tumour-infiltrating macrophages

| *Days after tumour transplant** | $EA_{50}$ *control* | $EA_{50}$ C. parvum *treated*** | *% Increase* |
|---|---|---|---|
| 13 | 305 | 380 | 25 |
| 14 | 322 | 448 | 39 |
| 16 | 241 | 588 | 144 |
| 17 | 333 | 650 | 95 |
| 17 | 292 | 500 | 71 |
| 17 | 294 | 500 | 70 |
| 17 | 277 | 385 | 39 |
| 17 | 185 | 286 | 55 |
| 21 | 223 | 428 | 92 |
| 21 | 294 | 422 | 44 |
| 22 | 339 | 550 | 62 |
| Mean (±s.e.) | 282 (±15) | 467 (±31)*** | 67 (±10) |

*5 x $10^5$ *in vivo* derived tumour cells inoculated s.c. on Day 0.
**0.25 mg *C. parvum* injected i.v. on Day 4.
***$P<0.001$ when the means of the $EA_{50}$ values are compared by Student's *t*-test.

**Figure 2** Velocity sedimentation fractionation of tumour infiltrating macrophages. a. Fc receptor avidity: (———) *C. parvum*; (-----) Control. b. (■-----■) % of EA rosette forming cells per fraction; (■———■) total cells per fraction $\times 10^{-4}$; (□———□) total EA rosette forming cells per fraction.

tumours were tested during the period of growth inhibition. This was largely restricted to those mφ present within the major host cell fraction containing cells sedimenting at 1 to 5.5 mm $h^{-1}$. In the experiment shown in Figure 2a, a less marked enhancement of FcR avidity was also found in the mφ sedimenting between 6 and 10 mm $h^{-1}$ but this increase was not a consistent finding in all *C. parvum*-treated tumours. Macrophages sedimenting in excess of 11 mm $h^{-1}$ were unaffected by *C. parvum* therapy.

### Discussion

The host cell content of immunogenic tumours undergoing *C. parvum*-induced growth inhibition does not differ significantly from progressor tumours growing in untreated syngeneic mice. *C. parvum*-induced tumour growth inhibition in this syngeneic system is however, T-cell dependent (McBride *et al.*, 1980) and thus one might expect some qualitative, if not a quantitative change, in those intra-tumour host cells representative of the cellular immune system. Changes in the activation state of tumour infiltrating mφ have been shown to be intimately associated with regression or progression of MSV tumours (Russell *et al.*, 1977) and in the present study we have demonstrated changes in this cell population to be associated with inhibition of growth of a chemically induced tumour following i.v. immunotherapy with *C. parvum*. Measurement of the activation state of unfractionated mφ isolated from regressor or progressor tumours indicated that when means of the two groups were compared a 67% increase in FcR avidity was found.

We have previously demonstrated that tumour infiltrating mononuclear phagocytes are

**Table III** Differential count on Day 14 fractionated control and *C. parvum* treated tumours

| | *Tumour cells* % | (*cells* × $10^4$) | *Macrophages* % | (*cells* × $10^4$) | *Lymphocytes* % | (*cells* × $10^4$) | *Polymorphonuclear neutrophils* % | (*cells* × $10^4$) |
|---|---|---|---|---|---|---|---|---|
| CONTROL | | | | | | | | |
| Unfractionated | 82 | | 14 | | 3 | | 1 | |
| I 1–5.5 mm $h^{-1}$ | 23 | (131) | 48 | (274) | 23 | (131) | 6 | (34) |
| II 5.5–8.5 mm $h^{-1}$ | 78 | (640) | 21 | (172) | 1 | (8.2) | 0 | (0) |
| III 8.5–11.5 mm $h^{-1}$ | 95 | (979) | 5 | (56) | | — | | — |
| IV 11.5–14 mm $h^{-1}$ | 97 | (412) | 3 | (22) | | — | | — |
| *C. parvum* TREATED | | | | | | | | |
| I 1–5.5 mm $h^{-1}$ | 33 | (249) | 40 | (302) | 26 | (196) | 1 | (8) |
| II 5.5–8.5 mm $h^{-1}$ | 82 | (1013) | 17 | (209) | 1 | (12) | 1.5 | (6) |
| III 8.5–11.5 mm $h^{-1}$ | 95 | (1064) | 5 | (51) | | — | | — |
| IV 11.5–14 mm $h^{-1}$ | 97 | (425) | 3 | (24) | | — | | — |
| Unfractionated | 82 | | 13 | | 4 | | 1 | |

heterogeneous with respect to FcR avidity (Moore & McBride, 1980). In that study we demonstrated the presence of two populations of m$\varphi$ exhibiting different activation levels of FcR and in the present study we determined which m$\varphi$ subpopulation was the target for *C. parvum* induced activation to gain some insight into how *C. parvum* was influencing m$\varphi$ differentiation within the tumour micro-environment.

When tumours from *C. parvum*-treated animals were subjected to fractionation by unit gravity velocity sedimentation no difference could be detected in the distribution of EA rosette forming cells when rosettes were formed using ORBC sensitised with high concentrations of antibody. However, when the m$\varphi$ were rosetted with ORBC sensitised with low levels of antibody, only the activated m$\varphi$ formed rosettes. This technique demonstrated that the smaller sized population of phagocytic cells within the tumour which sedimented at 1 to 5 mm $h^{-1}$ exhibited the greatest increase in activation state in response to *C. parvum* treatment. An eight-fold increase in FcR avidity was detected within this population in contrast to a mean 67% increase in the rapidly adherent non-fractionated m$\varphi$ preparations. This was caused by the presence within monolayers derived from non-fractionated cells of large, rapidly sedimenting m$\varphi$ which comprise ~50% of the total within the tumour. These m$\varphi$ are unaffected by *C. parvum* therapy and consequently their presence results in an apparent lowering of FcR avidity when heterogeneous monolayers are examined. Although cell monolayers analysed in these experiments were routinely >85% phagocytic it has to be considered that a minor non-phagocytic cell population expressing FcR that increase in avidity after *C. parvum* treatment may be responsible for the observed changes. Response to *C. parvum* treatment in this model is T-lymphocyte dependent (McBride *et al.*, 1980) and tumour infiltrating T-lymphocytes may be activated simultaneously with cells of the mononuclear phagocyte series. Activated T-lymphocytes (Yoshida & Anderson, 1972) and B-lymphocytes (Basten *et al.*, 1972) express FcR which would form rosettes under the conditions used, i.e. with ORBC sensitised with high concentrations of antibody if they did contaminate the m$\varphi$ monolayers. It is unlikely however, that the presence of these contaminating lymphocytes would contribute to the FcR avidity changes observed in the monolayers isolated from the slower sedimenting lymphocyte enriched fractions. Although activation of T-lymphocytes does increase FcR expression, this level is still 10 times less than that of proteose peptone-elicited peritoneal m$\varphi$ (Anderson & Grey, 1974). Earlier studies with the FSA/R tumour (Moore & McBride, 1980) indicated that the FcR avidity of the slower sedimenting tumour infiltrating m$\varphi$ in control animals was at least equal to that of proteose peptone-elicited m$\varphi$. In the present study we are measuring increases in excess of this level of FcR expression which eliminates the possibility of activated T-lymphocyte interference in the assay.

Results of our earlier study indicated that the intra-tumour phagocytic mononuclear cells sedimenting at the same velocity as those activated by *C. parvum* therapy, contain m$\varphi$ which are less differentiated than the larger more rapidly sedimenting cells and probably represents cells which have recently entered the tumour from the circulating monocyte pool. Systemic administration of *C. parvum* induces a generalised stimulation of the reticuloendothelial system (Baum & Breese, 1976) and monocytes may therefore be activated before extravasation to the solid tumour. We have previously demonstrated that full activation of m$\varphi$ is not achieved within the microenvironment of FSA/R tumours when growing progressively (Moore & McBride, 1980). The activation pathway of the intra-tumour m$\varphi$ in *C. parvum* treated mice may circumvent this problem by activating the m$\varphi$ at a site distant to the tumour. If intra-tumour m$\varphi$ differentiation is normally inhibited by the high concentration of suppressor factors at the tumour site (Spitalny & North, 1977) then *C. parvum* induced m$\varphi$ activation may be occurring where these factors are at an ineffective concentration. Thus activated monocytes/macrophages may enter the tumour where they are able to exert anti-tumour mechanisms before being inactivated.

This work was supported by a grant from the Cancer Research Campaign of Great Britain.

### References

ALEXANDER, P. (1976). The functions of the macrophage in malignant disease. *Ann. Rev. Med.*, **27**, 207.

ANDERSON, C.L. & GREY, H.M. (1974). Receptors for aggregated IgG on mouse lymphocytes: their presence on the thymocytes, thymus-derived, and have marrow derived lymphocytes. *J. Exp. Med.*, **139**, 1175.

BASTEN, A., MILLER, J.F.A.P., SPRENT, J. & PYE, J. (1972). A receptor for antibody on B lymphocytes. I Method of detection and functional significance. *J. Exp. Med.*, **135**, 610.

BAUM, M. & BREESE, M. (1976). Antitumour effect of *C. parvum*. Possible mode of action. *Br. J. Cancer*, **33**, 468.

EVANS, R. (1972). Macrophages in syngeneic animal tumours. *Transplantation*, **14**, 468.

GAUCI, C.L. & ALEXANDER, P. (1975). The macrophage content of some human tumours. *Cancer Letters*, **1**, 29.

KERBEL, R.S. & PROSS, H.F. (1976). Fc receptor-bearing cells as a reliable marker for quantitation of host lymphoreticular infiltration of progressively growing solid tumours. *Int. J. Cancer*, **18**, 432.

MANTOVANI, A. (1978). Effects on *in vitro* tumour growth of murine macrophages isolated from sarcoma lines differing in immunogenicity and metastasising capacity. *Int. J. Cancer*, **22**, 741.

MILLER, R.G. & PHILLIPS, R.A. (1969). Separation of cells by velocity sedimentation. *J. Cell Physiol.*, **73**, 191.

McBRIDE, W.H., PETERS, L.J., MASON, K.A. & BARROW, G. (1980). The effect of *C. parvum* on T-cell dependent tumour regression. *J. Reticuloendothelial Soc.*, **27**, 151.

MOORE, K. & MOORE, M. (1977). Intra-tumour host cells of transplanted rat neoplasms of different immunogenicity. *Int. J. Cancer*, **19**, 803.

MOORE, K. & McBRIDE, W.H. (1980). The activation state of macrophage subpopulations from a murine fibrosarcoma. *Int. J. Cancer*, **26**, 609.

MOORE, M. & MOORE, K. (1980). Intratumour host cells of experimental rat neoplasms: Characterisation and effector function. *Contemp Topics Immunobiol*, **10**, 109.

RUSSELL, S.W. & McINTOSH, A.T. (1977). Macrophages isolated from regressing. Moloney sarcomas are more cytotoxic than those recovered from progressing sarcomas. *Nature*, **268**, 69.

RUSSELL, S.W., GILLESPIE, G.V. & McINTOSH, A.T. (1977). Cytotoxicity mediated *in vitro* by macrophages recovered from disaggregated regressing Moloney sarcomas. *J. Immunol.*, **118**, 1574.

SPITALNY, G.L. & NORTH, R.J. (1977). Subversion of host defence mechanisms by malignant tumours: An established tumour is a privileged site for bacterial growth. *J. Exp. Med.*, **145**, 1264.

SUIT, H.D. & KASTELAN, A. (1970). Immunologic status of host and response of a methylcholanthrene induced sarcoma to local X-irradiation. *Cancer*, **26**, 232.

THOMSON, A.W., CRUICKSHANK, N. & FOWLER, E.F. (1979). Fc receptor-bearing and phagocytic cells in syngeneic tumours of *C. parvum*- and Carrageenan-treated mice. *Br. J. Cancer*, **39**, 598.

WOOD, G.W. & GOLLAHON, K.A. (1977). Detection and quantitation of macrophage infiltration into primary human tumours with the use of cell surface markers. *J. Nat Cancer Inst.*, **59**, 1081.

WOOD, G.W., NEFF, J.R., GOLLAHON, K.A. & GOURLEY, W.K. (1978). Macrophages in giant cell tumours of bone. *J. Pathol.*, **125**, 53.

YOSHIDA, T.O. & ANDERSON, B. (1972). Evidence for a receptor recognizing antigen complexed immunoglobulin on the surface of activated mouse thymus lymphocytes. *Scand. J. Immunol.*, **1**, 401.